# Fatigue characteristics in multiple sclerosis: the North American Research Committee on Multiple Sclerosis (NARCOMS) survey

**DOI:** 10.1186/1477-7525-6-100

**Published:** 2008-11-14

**Authors:** Olympia Hadjimichael, Timothy Vollmer, MerriKay Oleen-Burkey

**Affiliations:** 1 Department of Neurology, Yale University School of Medicine, New Haven, CT 06510, USA; 2 Barrow Neurological Institute, CMSC/NARCOMS Project, Phoenix, AZ 85013, USA; 3 Health Economics and Outcomes Research, Teva Neuroscience, Inc., Kansas City, MO 64131, USA

## Abstract

**Background:**

Fatigue is a common disabling symptom of multiple sclerosis (MS) and has a significantly negative impact on quality of life. Persons with MS enrolled in the North American Research Committee on Multiple Sclerosis (NARCOMS) Patient Registry are invited to complete follow-up surveys every six months to update their original registration information. One of these surveys was designed to focus on the severity and impact of fatigue, and its association with other clinical parameters of MS such as physical disability.

**Methods:**

In addition to the usual data collected in Registry update surveys such as demographic characteristics, MS-related medical history, disability and handicap, immunomodulatory and symptomatic therapies taken, and healthcare services used, the survey for this study included two validated self-report fatigue scales, the Fatigue Severity Scale (FSS) and the Modified Fatigue Impact Scale (MFIS) and questions about the use of symptomatic management for fatigue, both pharmacologic and non-pharmacologic treatments. This Registry update survey was mailed to all NARCOMS registrants (n = 18,595) in November 2002. Information provided by registry participants was approved for research purposes by the Yale University Institutional Review Board.

**Results:**

The response rate for the survey was 49.5% (9205/18,595). Severe fatigue as measured with the FSS using the developer's recommended severity cutpoint of ≥ 36 was reported by 6691 (74%) of evaluable respondents (n = 9077). A higher prevalence of severe fatigue was observed in relapsing-worsening MS compared with relapsing-stable and primary progressive MS. A distinct pattern of fatigue was observed across the disability levels of the Patient-Determined Disease Steps (PDDS). Although there were no differences in the severity or impact of fatigue by immunomodulatory agents (IMA), respondents who recalled therapy changes in the prior six months reported different patterns of change in fatigue with lower fatigue levels reported after changing from interferon-β to glatiramer acetate than after changing from glatiramer acetate to interferon-β. Concomitant therapy for fatigue was used by 47.2% of the 5799 survey respondents receiving IMA.

**Conclusion:**

Characterizing MS symptoms like fatigue can increase awareness about their impact on persons with MS and suggest recommendations for a care plan.

## Background

Chronic fatigue is one of the most common disabling symptoms among persons with multiple sclerosis (MS), interfering with, and considerably limiting, daily activities [1,2]. At least 65% of persons with MS experience fatigue on a daily basis, usually during the afternoons [3-6], and 15%–40% report it as the most disabling MS symptom [4,5,7-9]. MS fatigue is different from fatigue in healthy subjects [7,10], difficult to define, and therefore one of the most challenging symptoms to treat. No biologic or neuro-imaging markers for fatigue are currently known, and its pathophysiology and etiology are poorly understood. Both peripheral and central mechanisms may have a role [11-15].

Fatigue has a significant negative impact on daily work, family life, and social activities of persons with MS and is associated with the perception of an impaired general health, mental state, and quality of life (QOL) [16-18]. It appears to have an even more important effect on QOL than physical disability alone [18-20]. Studies of fatigue in association with other MS clinical characteristics, such as physical disability [8,21,22], depression [21,22], or disease subtype [4,8,9,21], report contradictory findings.

Symptomatic management of MS fatigue includes both non-pharmacologic treatments, such as exercise and keeping cool [1], as well as pharmacologic treatments, such as amantadine and modafinil [2].

The current study examined the characteristics of fatigue among persons with MS in the North American Research Committee on Multiple Sclerosis (NARCOMS) Patient Registry, a project of the Consortium of MS Centers (CMSC). A longitudinal database initiated in 1996, the Registry is a resource for clinical trials and long-term prospective studies [23-27]. As of 2008, the Registry is comprised of more than 33,000 patients and provides a unique opportunity to study MS characteristics and treatment patterns in a large population of persons with MS.

The aims of this study were to: (1) evaluate the severity and impact of fatigue among NARCOMS registrants and characterize the differences between those reporting mild/moderate fatigue and severe fatigue; (2) assess the association between the severity and impact of fatigue and physical disability; (3) investigate respondents' perceptions of fatigue levels when changing immunomodulatory agents (IMA); and (4) evaluate the prevalence and pattern of symptomatic management of fatigue.

## Methods

Persons with MS living in the US are recruited voluntarily to the NARCOMS registry through the registry's website, the National MS Society, MS centers, and support groups. The validity of the MS diagnosis was recently confirmed in 98.7 ± 1.3% of the validation sample [28]. Data collected in the registry include demographic information, MS-related medical history, disability and handicap, immunomodulatory and symptomatic therapies taken, and healthcare services used. Following enrollment, the Registry is updated with surveys that are sent to participants every 6 months [23,24]. The current study is based on a single Registry update survey that was mailed to all NARCOMS registrants (n = 18,595) in November 2002. Information provided by registry participants was approved for research purposes by the Yale University Institutional Review Board (IRB). The IRB granted approval for an information statement in lieu of formal informed consent. The Information Page accompanying each survey requested a participant's signature to acknowledge the intended use of the information and was worded as follows: "By signing below, I give my permission for the following information to be entered into the NARCOMS MS Registry. I understand that this information will be used for research purposes only, and that all responses will be kept private and confidential. I am willing to be notified of any studies for which I may be eligible."

This Registry update survey included a special section on fatigue. The most reliable and valid fatigue measures, the Modified Fatigue Impact Scale (MFIS) and the Fatigue Severity Scale (FSS) [4,29,30], were incorporated into the survey, along with a question designed to capture the registrants' perceptions of fatigue levels following changes from one IMA to another when that had occurred in the previous six months.

### Survey measures

#### MS-subtype

Participants in the NARCOMS registry are assigned a disease subtype based on the presence of relapses in the course of their disease and their disease progression. The first MS subtype, primary progressive disease, is defined as continuous accumulation of disability with no relapses throughout the disease course. Relapsing-stable disease is defined as having a relapse anytime throughout the disease course and reporting a disability state that is improved or similar to that observed a year earlier. Relapsing-worsening disease is defined as having a relapse anytime throughout the disease course and reporting a disability state that has worsened during the previous year.

#### Fatigue

The impact of fatigue on the respondent's daily activities was assessed with the MFIS, a 21-item scale that defines fatigue as a "feeling of physical tiredness and lack of energy that many people experience from time to time" [4,30]. Each item is rated by the respondent on a scale from 0 (never) to 4 (almost always). Scores are calculated for each of its three subscales: – physical (9 items, cumulative score range 0–36), cognitive (10 items, cumulative score range 0–40), and psychosocial (2 items, cumulative score range 0–8), and combined for a total MFIS score (range 0–84) [4,30].

Fatigue severity was measured with the FSS, a 9-item scale; each item is rated on a scale from 1 (strongly disagree) to 7 (strongly agree) with the total score ranging from 9–63 [29]. The scale developer defines severe fatigue as an FSS score ≥ 36 (an average of ≥ 4 across the nine questions) while mild/moderate fatigue is defined as FSS < 36 [29,31].

#### Mobility and neurologic impairment

Neurologic impairment and mobility status were assessed in the survey using two validated self-report instruments which are included in each Registry update survey.

Performance Scales are a measure of handicap in twelve domains of neurologic function: mobility, hand function, vision, fatigue, cognition, bladder/bowel, sensory, spasticity, pain, depression, tremor/loss of coordination, and anxiety. All domains except mobility are assessed by respondents on a scale of 0 (normal) to 5 (total disability). Mobility is measured on a 0 (normal) to 6 (total gait disability) scale [32].

Mobility impairment was measured with the Patient-Determined Disease Steps (PDDS), which uses a 0 (normal) to 8 (bedridden) scale [32,33]. The PDDS, although self-reported, is highly correlated with the physician-reported Kurtzke Expanded Disability Status Scale (EDSS) [34], and defines more precisely than the EDSS mid-range mobility. These scales were used to assess the correlation between PDDS and fatigue level, as well as the differences in fatigue levels based on the PDDS.

#### Fatigue and changes in IMA

The impact and severity of fatigue were examined relative to treatment with IMA along with the level of fatigue associated with changes in IMA. IMA included glatiramer acetate (Copaxone^®^) and the interferons: IFN-β-1a (Avonex^® ^and Rebif^®^), and IFN-β-1b (Betaseron^®^). Fatigue associated with therapy change was assessed with a scale of 1 (much less) to 7 (much greater), with a higher score indicating a higher level of fatigue following the change in therapy. A regression analysis was conducted to assess factors that could contribute to a change in fatigue under these circumstances.

#### Symptomatic treatments of fatigue

The use of symptomatic treatments for fatigue, both pharmacologic and non-pharmacologic, was collected in the survey. Non-pharmacologic treatment options included the use of an exercise program and physical and occupational therapy.

### Statistical analysis

Statistical analysis was performed using descriptive statistical techniques. Logistic regression was used to determine odds ratio estimates of the strength of the association between each dichotomous independent variable and the fatigue score, after controlling for the other variables in the model (age, use of symptomatic drugs, PDDS, duration of IMA use, disease duration, and disease subtypes). In addition, a separate regression analysis was conducted to assess factors that may contribute to changes in fatigue level after changes in IMAs.

## Results

The surveys were completed by 9205 (49.5%) registrants. To be evaluable, the survey had to have all fatigue questions answered and the respondent had to report treatment with an IMA or be treatment-naïve (n = 9077). The registrants who did not respond to the survey were similar to the respondents in mean age, gender distribution, disease duration, and education. The majority (65%) of the non-responders had been classified as having relapsing-worsening MS on the last completed Registry survey, and their mean PDDS score was 3.78 (early cane).

### Fatigue severity and impact

On the basis of the FSS scores, respondents were categorized into two levels of fatigue: mild/moderate (< 36) or severe (≥ 36) fatigue. Nearly 74% of the sample reported severe fatigue. As shown in Table 1 those with severe fatigue were more likely to be older, male, with an education level of associate degree or less, unemployed, diagnosed at an older age, and report more disability on the PDDS (*p *< .0001) than those with mild/moderate fatigue. Only 29.0% of those with severe fatigue reported being employed compared to 54.6% of those with mild/moderate fatigue. The level of fatigue also differed between the MS disease subtypes. A higher prevalence of severe fatigue was observed in persons with relapsing-worsening MS (59.8%) than among the other two subtypes. More respondents with severe fatigue (46.5%) were treated with symptomatic drugs than those with mild/moderate fatigue (18.2%).

**Table 1:** Demographic and MS characteristics among NARCOMS respondents by fatigue severity^a^

	**Mild/Moderate Fatigue** **(N = 2386)**	**Severe Fatigue** **(N = 6691)**	**P value**
**Demographic Characteristics**			

Mean age, years (± sd)	45.8 (± 11)	48.3 (± 10)	<.0001

Gender: female, %	75.8	70.6	<.0001

Race: Caucasian, %	92.6	92.9	

Education: associate or less, %	52.0	61.5	<.0001

Employed, %	54.6	29.0	<.0001

**MS Characteristics**			

Mean age at diagnosis, years (± sd)	36.5 (± 9.6)	38.0 (± 9.6)	<.0001

MS duration (years since diagnosis) (± sd)	12.1 (± 9.5)	13.1 (± 9.6)	

Disability: mean PDDS score	2.58	4.22	<.0001

MS types, %			<.0001

Relapsing-stable	59.6	32.6	

Relapsing-worsening	26.5	59.8	

Primary progressive	14.0	7.6	

Treatment with symptomatic drugs for fatigue, %	18.2	46.5	

PDDS, Patient-Determined Disease Steps (disability scale range: 0–8)

^a^mild/moderate fatigue = FSS score <36; severe fatigue= FSS score ≥ 36

The impact of fatigue as measured with the MFIS was notably higher among those with severe fatigue as shown in Table 2. The mean total MFIS score as well as each of the mean MFIS subscale scores for physical, cognitive, and psychosocial fatigue were more than twice as high among those with severe fatigue as among those with mild/moderate fatigue. Correspondingly, respondents with relapsing-worsening disease who were shown to have the most severe fatigue also had a higher mean total MFIS score (51.3 ± 15.9)compared to those with relapsing-stable (36.8 ± 18.3) and primary progressive (36.9 ± 18.9) MS.

**Table 2:** Mean fatigue scores by fatigue severity in NARCOMS respondents^a^

**Fatigue scale**	**Mild/Moderate Fatigue-mean score (± sd)** **(N = 2386)**	**Severe Fatigue-mean score (± sd)** **(N = 6691)**
MFIS^b^		

Total MFIS (± sd)	24.5 (± 14.5)	51.1 (± 14.4)

Physical (± sd)	12.5 (± 7.4)	25.7 (± 6.2)

Cognitive (± sd)	9.8 (± 7.5)	20.3 (± 8.8)

Psychosocial (± sd)	2.2 (± 1.8)	5.2 (± 1.8)

FSS		

Total FSS (± sd)	23.7 (± 8.8)	52.4 (± 7.7)

MFIS, Modified Fatigue Impact Scale; FSS, Fatigue Severity Scale.

^a^Mild/moderate fatigue = FSS score <36; severe fatigue = FSS score ≥ 36

^b^Score ranges = MFIS: Total MFIS 0–84, MFIS physical subscale 0–36, MFIS cognitive subscale 0–40, MFIS psychosocial subscale 0–8; FSS 9–63

When mean fatigue scores on the MFIS and FSS were examined relative to the duration of MS, they were shown to sharply increase for about the first 14 years of disease duration, after which they leveled off in terms of both impact (MFIS) and severity (FSS).

A logistic regression analysis of several factors thought to predict fatigue among the NARCOMS respondents showed that the use of symptomatic drugs for fatigue was a strong predictor while PDDS was a weak predictor for experiencing fatigue (Table 3). Having relapsing-stable or primary progressive MS rather than relapsing-worsening MS was predictive of lower fatigue. Age, duration of IMA therapy, and disease duration showed no predictive power.

**Table 3:** Predictors of fatigue among NARCOMS respondents

	**Odds Ratio**	**95% CI**
Use of symptomatic drugs	3.85	3.26, 4.55

PDDS	1.38	1.32, 1.43

Age	1.02	1.01,1.03

IMA duration	0.996	0.99, 0.99

Disease duration	0.978	0.97, 0.99

Relapsing-stable vs. Relapsing- worsening MS type	0.434	0.37, 0.51

Primary-progressive vs. Relapsing-worsening MS type	0.233	0.18, 0.31

IMA, immunomodulatory agent; PDDS, Patient-Determined Disease Steps

### Fatigue and MS disability

**Figure 1:**
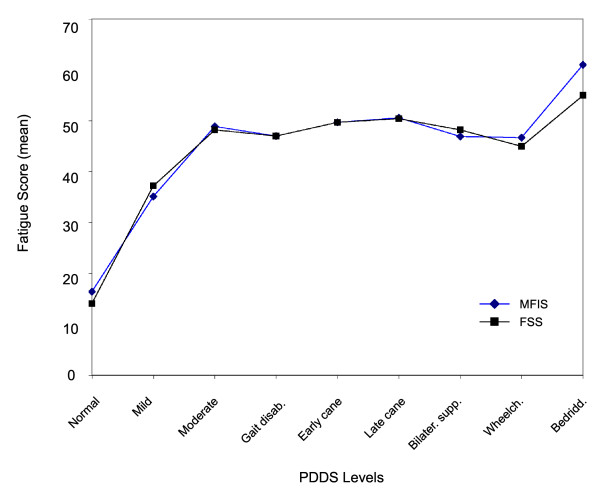
**Fatigue by PDDS levels**. FSS, Fatigue Severity Scale; MFIS, Modified Fatigue Impact Scale; PDDS, Patient-Determined Disease Steps.

Mean fatigue scores measuring impact (MFIS) and severity (FSS) were found to follow similar patterns across the levels of physical disability on the PDDS (Figure 1). Fatigue scores increased steadily as respondents' functional levels changed from no limitations, to abnormal gait. From that point on up to wheelchair mobility, fatigue impact and severity remained stable. However, respondents who were bedridden reported the most severe fatigue, on average, of any disability category and the impact of that fatigue as measured by the MFIS was also at its highest point.

Neurologic impairment, as reflected in the mean scores of all twelve domains of the Performance Scales, including depression, was consistently statistically significantly higher (*p *< .0001) in respondents with severe fatigue compared with those reporting mild/moderate fatigue. The following scores show the differences between severe fatigue and mild/moderate fatigue, respectively, in these Performance Scale domains: fatigue (3.2 ± 1.1 vs. 1.3 ± 1.0), mobility (3.3 ± 1.8 vs. 2.1 ± 2.2), hand function (1.8 ± 1.9 vs. 1.0 ± 1.2), vision (1.5 ± 1.2 vs. 0.9 ± 1.0), cognition (1.9 ± 1.3 vs. 0.9 ± 0.9), bladder function (2.1 ± 1.4 vs. 1.2 ± 1.2), sensory (2.1 ± 1.4 vs. 1.1 ± 0.9), spasticity (2.1 ± 1.4 vs. 1.1 ± 1.1), pain (1.9 ± 1.4 vs. 0.9 ± 1.0), and depression (1.6 ± 1.2 vs. 0.7 ± 0.8).

### Fatigue and use of IMA

**Figure 2:**
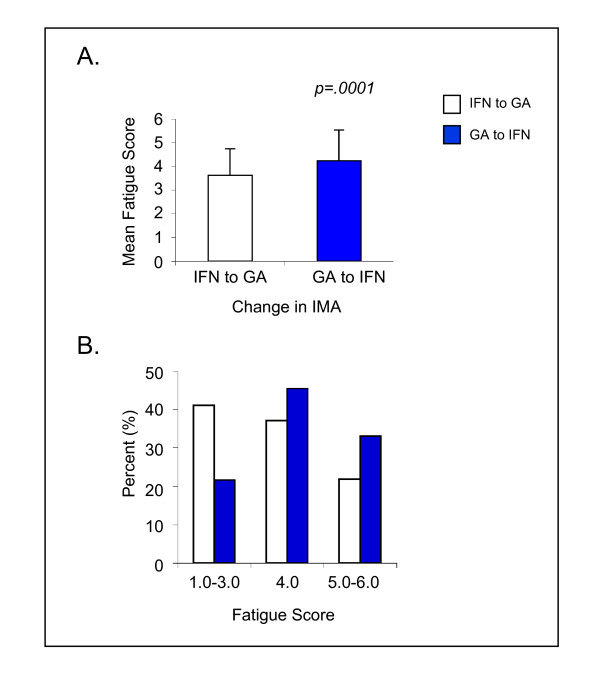
**Changes in fatigue rating following a change in IMA**. NARCOMS respondents who changed from IFN to GA (n = 766) and from GA to IFN (n = 218). IFN, Interferon β-1a or 1b; GA, glatiramer acetate; IMA, immunomodulatory agents.

Among the respondents, 5805 (64.0%) were being treated with IMA which are often recommended and prescribed for MS to reduce relapse rates and slow the accumulation of disability: 3720 (41.0%) were treated with various interferons, 2085 (23.0%) with glatiramer acetate (GA), and 324 (3.6%) with other therapies, such as azathioprine and gamma globulin. Cross-sectional response to the fatigue questionnaires did not show any statistically significant differences in severity or impact relative to the treatments; however, level of fatigue was found to be different when patients recalled a time of therapy change in the past six months. Fatigue levels following a change in therapy from an interferon (IFN) to glatiramer acetate (GA) or from GA to an IFN were compared to fatigue levels prior to the therapy change. The 766 respondents who reported changing from IFN to GA therapy reported significantly lower fatigue levels compared to the 218 respondents who reported changing from GA to IFN (3.6 vs 4.2, *p *= .0001) (Figures 2A). Similarly, at the time of the survey, there was a higher percentage of respondents with low fatigue scores among those who had changed from IFN to GA than among those who had changed from GA to IFN (Figure 2B). Regression analyses confirmed that changing from IFN to GA contributed to a decrease in fatigue (*p *< .0001, parameter estimate = -0.66).

### Symptomatic treatments for fatigue

**Figure 3:**
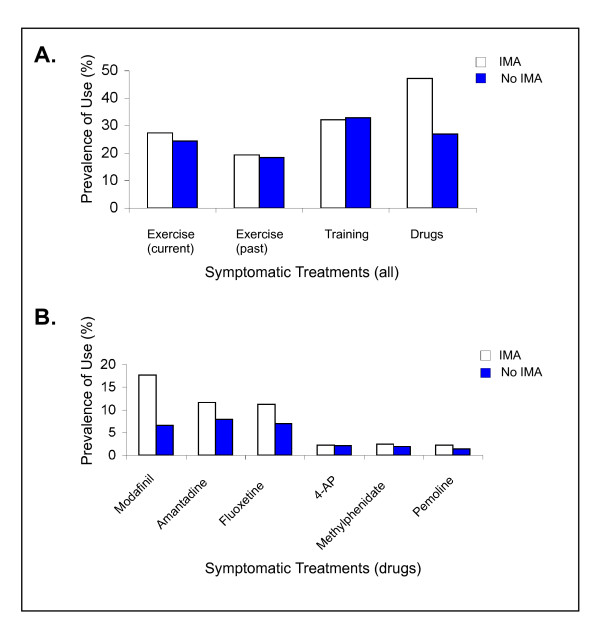
**Current use of symptomatic treatments for fatigue based on the use of IMA**. IMA = NARCOMS respondents treated with IMA;No IMA = NARCOMS respondents not treated with IMAs. 4-AP, 4-aminopyridine; IMA, immunomodulatory agents.

Among the respondents treated with IMA, 96.6% received symptomatic treatments for fatigue and among those untreated with IMA 95.6% received symptomatic treatment. Non-pharmacologic approaches showed a similar pattern of use in respondents treated and not treated with IMA: approximately 27% reported participating in an exercise program and 33% received physical and occupational therapy for their fatigue (Figure 3A). However, the use of pharmacologic agents for fatigue differed between the two groups: 47.2% of respondents receiving IMA reported the concurrent use of at least one of the symptomatic drugs for fatigue listed in the survey (Figures 3A and 3B). The most frequently used was modafinil (17.6%), followed by amantadine (11.6%), fluoxetine (11.2%), and others (6.8%). Among 3076 respondents who were not treated with IMA, a lower overall prevalence of pharmacologic treatment for fatigue (26.9%) and a different pattern of use was observed: amantadine (7.9%), fluoxetine (7.0%), modafinil (6.6%), and others (5.4%) (Figure 3B).

## Discussion

Our survey of the NARCOMS registrants shows a high prevalence of severe fatigue (74%) among persons with MS that impacts activities of daily living as measured with the FSS and MFIS. Smaller studies have previously reported fatigue in relation to MS measured by various fatigue instruments [5,8-10,21,22,35,36], but the current study is the largest to date.

Respondents with severe fatigue differed from respondents with mild/moderate fatigue on a variety of factors including employment status, physical disability level, and disease subtype. Even though employment is valued both for economic reasons and for reasons associated with identity, self-esteem, and social contact, the low employment rate among those with severe fatigue is consistent with earlier reports that fatigue is a major cause of early retirement and unemployment in persons with MS [37-39].

Respondents with severe fatigue also had significantly higher mobility impairment as measured by PDDS scores compared with respondents with mild/moderate fatigue. When fatigue was evaluated for persons at the various levels of PDDS, it was found to increase sharply with increasing mobility impairment prior to gait disability, then leveled off until the bedridden stage when it again increased sharply. The PDDS scores in the current study were modestly predictive of fatigue (OR= 1.38). Other studies have shown a positive association between fatigue severity and the EDSS, which is highly correlated with the PDDS [32]. In some cases the positive association between EDSS and fatigue was seen with no adjustment for other confounding factors while in other cases the association was found after an adjustment for depression, duration of disease, or age [8,9,14,21,35,40]. Conversely, there are studies that do not show any correlation between fatigue and the EDSS [4,5,10]. Our use of a large sample of persons with MS who reported a wide spectrum of PDDS levels may have enabled us to uniquely observe the full pattern of fatigue across the entire disease course of MS.

Previous reports suggest that fatigue levels in primary progressive disease are higher than those in other subtypes while our results show that among NARCOMS survey respondents, the highest level of fatigue was reported in the relapsing-worsening subtype, and those with primary progressive disease reported the least fatigue [8,9,16,40]. Kroencke et al. have shown that persons with both primary and secondary progressive MS have higher fatigue levels than persons with relapsing-remitting MS, and attribute this finding to the differences in disability among the three disease subtypes [21]. In this study, we have shown that those with relapsing-stable MS have much less fatigue than those with relapsing-worsening disease and this may be associated with more active disease in the latter subtype.

The current study showed that age and disease duration were not predictors of fatigue which is consistent with earlier published reports [21,22,36]. However, respondents with severe fatigue were somewhat older (a mean difference of 2 years) than those with mild to moderate fatigue. In terms of disease duration, fatigue increased steadily for people with MS durations of one to 14 years, but for those with longer durations of MS there was a leveling off of fatigue.

In this cross-sectional look at fatigue among people with MS using the commercially available IMA, there was no significant difference in either severity or impact of fatigue. This finding is inconsistent with a previous report where patients beginning therapy with IMA were evaluated for their fatigue levels after 6 months of therapy, and 25% of those receiving GA therapy had significantly improved fatigue compared to only 12% of IFN users [41]. These results may be different because we looked at fatigue among patients who were at all stages of using IMA, not just those within the first six months of beginning therapy. In contrast, among respondents who reported changing IMA within the past six months, significantly lower fatigue levels were recalled among respondents who changed from IFN-β to GA as compared with respondents who stopped GA and began IFN-β. Because therapy change is often related to worsening of disease, there may have been more fatigue among those who experienced a change in therapy and any improvement or worsening of fatigue would have been particularly noteworthy. Additional factors may be physiological: it has been reported that an increase in proinflammatory cytokines may be a possible contributor to primary fatigue in MS [40,42]. GA has been shown to induce a shift from Th1 to Th2 response, resulting in lower levels of these cytokines [43-45] which correlate with a clinical response to GA [46]. In addition, treatment with IFN-β may produce secondary fatigue in MS in conjunction with an initial adverse effect of flu-like symptoms [47,48].

Fatigue is one MS symptom that is under-treated from a pharmacologic perspective: a recent survey among veterans with MS showed that only 40% of the people who reported having fatigue received pharmacological agents for treating fatigue [49], and in an Italian study of 856 persons with MS, fatigue was the symptom most frequently untreated with pharmacologic agents [50]. However, due to the high impact of fatigue on their ability to carry out their usual activities as well as their QOL, the use of various treatment modalities, both non-pharmacologic and pharmacologic is warranted. In the current study, less than a quarter of the respondents used non-pharmacologic means, such as conserving energy and exercising, to deal with their fatigue. Those with severe fatigue reported higher use of symptomatic drugs, as expected, and respondents using IMA reported a higher prevalence (47.2%) of use compared to those not being treated with IMA (26.9%). Persons with MS who are not being treated with IMA may have less symptoms overall, including fatigue, which could account for their lower level of pharmacologic treatment. While no drugs are currently FDA approved for the symptomatic treatment of MS fatigue, several drugs, such as amantadine, modafinil, pemoline, and 4-aminopyridine, have been shown to provide benefit [15,35,50,51]. The guidelines for fatigue management developed by the MS Council suggest amantadine as the first-line therapy and pemoline as a second-line agent [2]. Surprisingly, fluoxetine, an antidepressant, was commonly used for fatigue among NARCOMS respondents, although no clinical trials have proven its efficacy in MS fatigue treatment.

This study provides a potential benchmark for the pattern of fatigue severity and impact across the MS disease course. Strengths of this study include the size of the respondent sample, and the broad spectrum of mobility impairment it represents. It assessed fatigue with scales that have been well validated in MS. As with any research, however, it is important that the findings be interpreted in the context of the limitations of the study design: All scales used in the current NARCOMS update survey relied on respondent perceptions. Although self-report surveys are currently the most widely used instruments for fatigue evaluation in MS, objective measures of fatigue have occasionally been used in conjunction with surveys to bring another dimension to the symptom [17,52], and that was not done for this study. The analyses of the fatigue measures also did not control for depression which has been shown by some investigators to be associated with fatigue. While depression may be influencing the number of patients reporting severe fatigue, especially among the relapsing-worsening MS subtype, it is also possible that some misclassification of disease subtype occurred between relapsing-worsening and primary progressive subtypes that contributed to more severe fatigue in the relapsing-worsening category. Another possible limitation may be the 50% response rate which is generally considered adequate for surveys of this nature. Since data for non-responders from the registry showed that they were similar in demographic characteristics to the respondents, but included more patients with relapsing-worsening MS, our results may underestimate the proportion of MS patients who experience severe fatigue.

## Conclusion

The results of our study suggest that due to its high prevalence and impact on daily activities including employment, fatigue should be evaluated routinely and pharmacologic and non-pharmacologic treatments recommended for an MS care plan.

## Competing interests

OH and TV have no competing interests. MOB is an employee of Teva Neuroscience, Inc., which funded this research project.

## Authors' contributions

OH was involved in study design, data collection and analysis, manuscript planning and editing. TV and MOB were involved in study design, manuscript planning and editing. All authors read and approved the final manuscript.
